# *Ab initio* structure determination of nanocrystals of organic pharmaceutical compounds by electron diffraction at room temperature using a Timepix quantum area direct electron detector

**DOI:** 10.1107/S2053273315022500

**Published:** 2016-02-05

**Authors:** E. van Genderen, M. T. B. Clabbers, P. P. Das, A. Stewart, I. Nederlof, K. C. Barentsen, Q. Portillo, N. S. Pannu, S. Nicolopoulos, T. Gruene, J. P. Abrahams

**Affiliations:** a Biophysical Structural Chemistry, Leiden University, Einsteinweg 55, 2333 CC Leiden, The Netherlands; b Center for Cellular Imaging and NanoAnalytics (C-CINA), Biozentrum, University of Basel, CH-4058 Basel, Switzerland; c Nanomegas SPRL, Boulevard Edmond Machtens 79, B 1080, Brussels, Belgium; d Department of Physics and Energy, Materials and Surface Science Institute (MSSI), University of Limerick, Limerick, Ireland; e Amsterdam Scientific Instruments, Postbus 41882, 1009 DB Amsterdam, The Netherlands; f Centres Científics i Tecnològics de la Universitat de Barcelona, University of Barcelona, Carrer de Lluís Solé i Sabaris, 1-3, Barcelona, Spain; g Biology and Chemistry, Laboratory of Biomolecular Research, Paul Scherrer Institute (PSI), 5232 Villigen, Switzerland

**Keywords:** electron nanocrystallography, Timepix quantum area detector, carbamazepine, nicotinic acid, electron diffraction structure determination

## Abstract

Until recently, structure determination by transmission electron microscopy of beam-sensitive three-dimensional nanocrystals required electron diffraction tomography data collection at liquid-nitrogen temperature, in order to reduce radiation damage. Here it is shown that the novel Timepix detector combines a high dynamic range with a very high signal-to-noise ratio and single-electron sensitivity, enabling *ab initio* phasing of beam-sensitive organic compounds. Low-dose electron diffraction data (∼0.013 e^−^ Å^−2^ s^−1^) were collected at room temperature with the rotation method. It was ascertained that the data were of sufficient quality for structure solution using direct methods using software developed for X-ray crystallography (*XDS*, *SHELX*) and for electron crystallography (*ADT3D*/*PETS*, *SIR2014*).

## Abstract 2

A specialized quantum area detector for electron diffraction studies makes it possible to solve the structure of small organic compound nanocrystals in non-cryo conditions by direct methods.

## 1. Introduction   

Electron diffraction (ED) is a widely used tool for the determination of crystal structures of inorganic materials and metals (Williams & Carter, 2009 ▸). As electrons are less damaging than X-rays by several orders of magnitude relative to the amount of energy that is absorbed (Henderson, 1995 ▸), ED should be an attractive alternative to X-ray diffraction, when only nanometre-sized crystals^1^ of radiation-sensitive molecules are available. However, difficulties with sample preparation and beam damage have remained major bottlenecks in transmission electron microscopy (TEM) studies of pharmaceutical compounds and other beam-sensitive mater­ials. In X-ray crystallography, data are acquired by rotating the crystal during exposure about a constant axis normal to the direction of the beam. This method (known as ‘the rotation method’) has been the standard approach in protein X-ray crystallography for the last 40 years. It ensures good coverage of reciprocal space, it is experimentally less demanding than other approaches to data collection, it is optimal for radiation-sensitive crystals and it allows straightforward data processing (Arndt & Wonacott, 1977 ▸). The rotation method has more recently been adopted in the field of electron crystallography (Nederlof *et al.*, 2013 ▸; Nannenga *et al.*, 2014 ▸; Gemmi *et al.*, 2015 ▸), where it has become known as ‘automated diffraction tomography’ (ADT) (Kolb *et al.*, 2007 ▸, 2008 ▸; Mugnaioli *et al.*, 2009 ▸). This and related methods like rotation electron diffraction (RED) (Zhang *et al.*, 2010 ▸; Wan *et al.*, 2013 ▸; Yun *et al.*, 2015 ▸) have been used to solve crystal structures of beam-sensitive compounds *ab initio* from three-dimensional ED data acquired at low electron dose from cryo-cooled samples. There are examples of beam-sensitive materials solved at ambient temperature by ED, but these structures were solved using the non-continuous ADT technique, *e.g.* benzamides (Gorelik *et al.*, 2012 ▸) and 9,9′-bianthracene-10-carbonitrile (CNBA) (Kolb *et al.*, 2010 ▸), and used a higher electron dose than reported here (more than 100 e^−^ Å^−2^).

Pharmaceutical compounds can exist in different crystal packing forms called polymorphs. Different polymorphs can have different physical properties, like dissolution rate in water, stability or bioavailability. Pharmaceutical compounds can often exist as multiple polymorphs and each one is separately patentable, so obtaining a polymorph portfolio of a specific pharmaceutical compound can also be of great economic value. To determine polymorphic crystal structures, both single-crystal and powder X-ray diffraction can be used. For crystals of 0.5 µm or larger, single-crystal X-ray diffraction is adequate, but for smaller crystals beam damage becomes an issue. For such samples, X-ray powder diffraction is used. Since in X-ray powder diffraction the signal of a large number of crystals is averaged, the signal of individual nanocrystals will be lost in the signal of larger crystals. So this method may fail to detect one or more polymorphic modifications associated only with nanocrystals, especially if they are present in small amounts (*e.g.* less than 1–5%wt). However, an ED study using a standard transmission electron microscope may reveal the structure of any individual nanocrystal from 20 nm to several µm in size. So far, structure determination by ED of three-dimensional nanocrystals of beam-sensitive organic crystals has required data collection at liquid-nitrogen temperature, in order to reduce radiation damage (Eddleston *et al.*, 2010 ▸). There are multiple reasons to consider doing experiments at ambient temperature when possible: (i) interference of ice crystals by ice formation during sample load; (ii) necessity of in-column cryo-plates to reduce contamination; (iii) possible change in crystal structure; (iv) reduced sample throughput, as loading/unloading samples can take up to 2 h.

Hybrid pixel quantum area detectors [*e.g.* the Dectris single photon counting detectors (Brönnimann *et al.*, 2002 ▸)] have revolutionized X-ray crystallography.

The Medipix detectors that have been – and continue to be – developed by CERN-led consortia (Llopart *et al.*, 2002 ▸, 2007 ▸) have also been tested for electron detection (*e.g.* McMullan *et al.*, 2009 ▸; Georgieva *et al.*, 2011 ▸). They have much improved characteristics compared to CCD cameras often used to collect ED data. CCD cameras have several drawbacks for efficient ED data collection, namely: (i) blooming effects, where bright intense areas overshadow weaker ones located nearby; (ii) the need to use a beamstop to prevent damage to the camera; (iii) low signal-to-noise ratio (S/N); (iv) camera dark current; (v) increased background due to sensitivity to X-rays (abundantly produced in a transmission electron microscope). Such drawbacks negatively influence the quality of ED data that can be collected from radiation-sensitive samples, from organic pharmaceutical compounds to proteins.

Here, we describe the benefits of the Timepix detector. The Timepix, which is a member of the Medipix family of detectors, is able to count only those particles that have an energy that is higher than a user-defined, programmable threshold. As the Timepix detector can discriminate between different types of quanta based on their energy, the noise signal from the abundantly present lower-energy X-rays is filtered out. As a consequence, the detector has a very high S/N. Also dark current, which is present in CCD cameras due to thermal energy accumulation adding to static noise, is non-existent in the Timepix detector. At higher electron energies the corresponding scattering of the primary electron in the silicon detective layer can lead to charge sharing, resulting in counts in neighbouring pixels other than the location of impact; this only leads to a slight spreading of the Bragg spots. Next to the very high S/N, the chip has a high linear range, a dynamic range of 11.8k electron counts with a linear range of 10k electron counts per acquired frame. Therefore very weak Bragg spots are visible next to very bright Bragg spots. These characteristics make the Timepix detector an ideal tool for quantitative diffraction. We investigate how Timepix would facilitate structure determination of radiation-sensitive three-dimensional nanocrystals without having to cryo-cool the sample.

## 2. Methods   

### 2.1. Camera   

**Figure 1:**
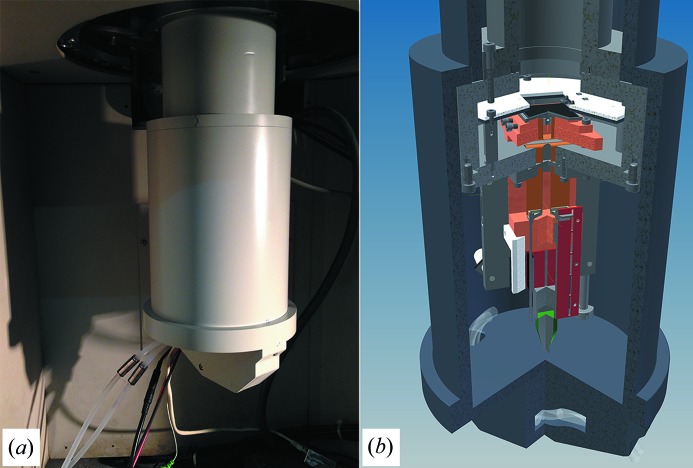
(*a*) Detector mounted at the on-axis camera position on a CM30 (University of Barcelona, CciTUB services, Spain). (*b*) 90° cut-off schematic of the camera as designed for the CM200 (Technische Universiteit Delft, The Netherlands).

**Figure 2:**
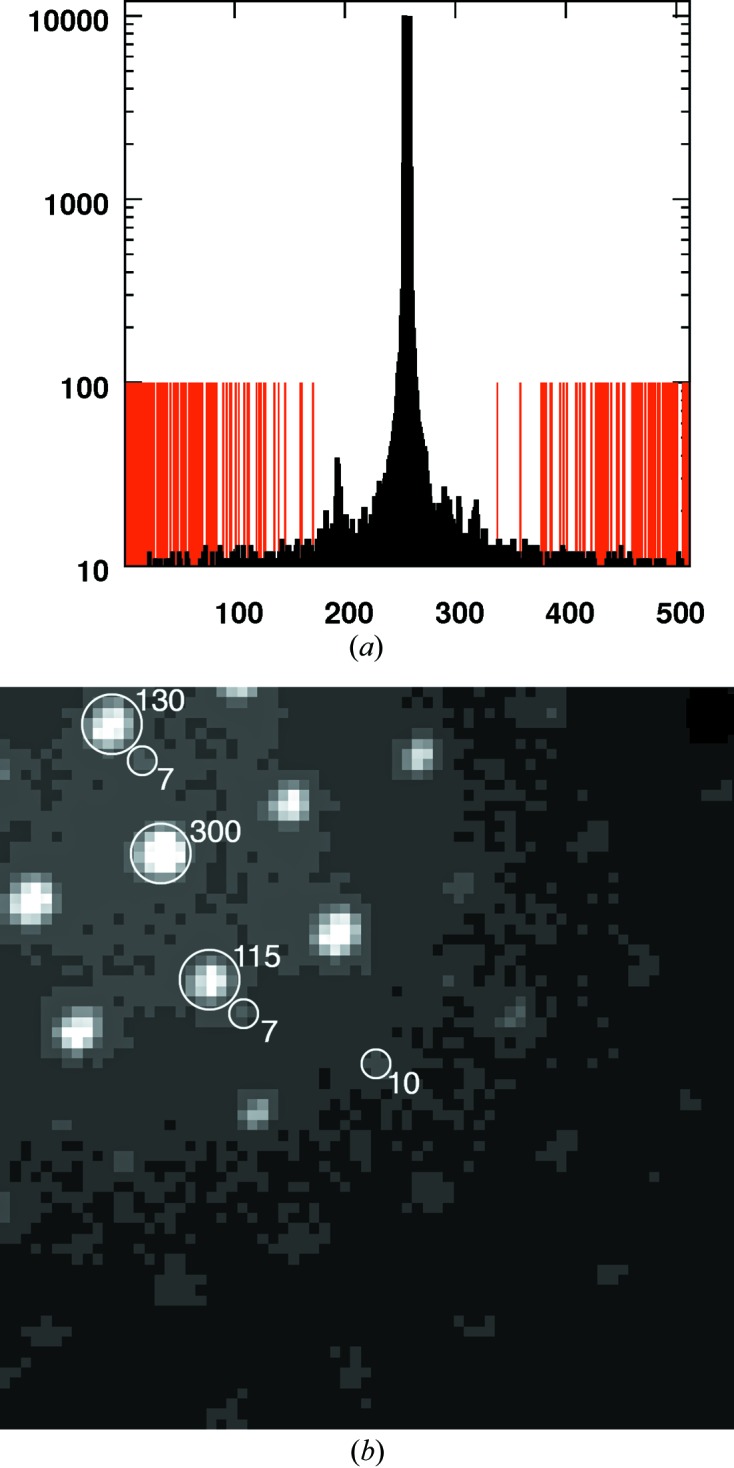
(*a*) Linear cross section through an image recorded with the Timepix detector ranges from 0 counts (marked red, set to 100 for visibility) to 10 020. Note the absence of a beamstop! (*b*) Spot separation of closely neighbouring spots in the absence of ‘blooming’ effects.

The camera^2^ we developed is modular and was fitted onto different transmission electron microscopes, using different adaptor flanges. Up to now it has been tested on the following microscopes: Philips-FEI CM30 (LaB6), CM200 (FEG), CM300 (FEG), Zeiss Libra 120 (LaB6). The Timepix detector chip assembly, its Relaxed readout electronics board and readout and control software were provided by Amsterdam Scientific Instruments (ASI). The Timepix chip was designed at CERN under the guidance of the Medipix collaboration (Llopart *et al.*, 2007 ▸). The Timepix detector we used consists of four single Timepix chips which were bump-bonded to a silicon sensor of 300 µm. The thickness of 300 µm is sufficient to capture all the energy of 200 keV electrons while preventing damage to the Timepix ASIC (McMullan *et al.*, 2009 ▸). A single Timepix chip has 256 by 256 pixels and a pixel size of 55 µm, with larger pixels of 175 µm on the edges. Therefore, a quad assembly has gaps between the individual four chips of twice 175 µm. This leads to a higher count at pixels 256 and 257, which needs to be taken into account. Combining the four chips gives a total pixel count of 512 by 512 pixels. The detector was built into a vacuum pod and is actively thermostated to 293 ± 0.1 K (Fig. 1 ▸). The Relaxed readout board, which can reach a frame rate of 120 fps (frames per second) (Visser *et al.*, 2011 ▸), was outside the vacuum. The detector was calibrated using the *SoPhy* software from ASI, in order to ensure a uniform energy threshold for triggering each pixel. The cross section of an example diffraction frame in Fig. 2 ▸(*a*) includes the direct beam position. It contains the dynamic range from zero counts to 10 020 on one frame. The edges contain continuous stretches of zero counts, *i.e.* the noise is due to scattering and the Timepix detector has no readout noise or dark current. The absence of ‘blooming’ effects enables spot separation of weak peaks close to strong peaks, illustrated in Fig. 2 ▸(*b*).

### 2.2. Microscope   

A Philips CM30 LaB6 microscope (University of Barcelona, CciTUB services) was operated in standard TEM mode at 200 kV. The very low dose rate was acquired by using the smallest available condenser aperture (20 µm), an extraction voltage step of 29 and spot size 9. This resulted in a dose of 0.013 e^−^ Å^−2^ s^−1^. The beam was aligned to be as parallel as possible. This was achieved on the fluorescent screen at higher beam intensities, then slowly the spot size was lowered to 9 while keeping the beam shape and angle as constant as possible.^3^ The dose rate was measured by lowering the frame acquisition time to 0.1 ms. This way, individual electron impacts were recorded; these were counted and the dose rate was interpolated. The single tilt rotation holder allowed rotation of the stage from −60° to 60°. The stage was tested and the tilt speed was linear at speeds below 1° per second for all angles. The semi-automatic alpha tilt stage required the operator to read the rotation angle directly from the stage providing a precision of 0.3°. The camera was operated with an ‘exposure time’ of 0.1 s, including dead time. This resulted in an acquisition rate of 9.1 fps.

### 2.3. Sample preparation   

Nicotinic acid was obtained from Carlo Erba Reagents, CAS: 59-67-6. Carbamazepine was kindly provided by Crystallics (Amsterdam). Nanocrystalline samples were obtained by crushing dry crystals into a fine powder by rubbing them between two glass microscope slides. The powder was then carefully applied several times on a holey carbon 300 mesh microscope grid. The powder on different areas of the glass slides was applied on separated parts of the grid, in order to transfer a small amount of nanocrystals (from 50 to 500 nm in size) with random size distribution and orientation onto the electron microscope grid.

### 2.4. Data collection   

Data were collected at 200 keV. Once a suitable crystal was found in TEM imaging mode at 5.3k magnification, it was centred by adjusting the *Z* height until the crystal remained at the same *xy* position whilst tilting the holder. Data were collected by continuous rotation of the crystal (Arndt & Wonacott, 1977 ▸). The maximum tilt range was found manually by ensuring that the crystal remained in the beam over the entire observed tilt range. This range usually varied from 15° to 60°, depending on stage stability and the sample holder. For data collection, the microscope was switched to diffraction mode and the corresponding α angle was recorded; when the maximum tilt angle was reached (where either the crystal was tilted out of the beam or the maximum tilt angle of the holder was reached) the corresponding final α angle was recorded. ED frames were recorded during the stage rotation with the Timepix detector and data were saved as binary images to be further processed. Collection of a full rotation data set required a few minutes (3–6 min) at most.

### 2.5. Data pre-processing   

Data sets from rotation ED patterns were pre-processed for further analysis. Pre-processing (using software developed in-house) included: (i) correction for the spatial distortion and larger size of pixels at the horizontal and vertical central axes of the Timepix quad; (ii) correction for any dead or bright pixels; (iii) centring of the images on the direct beam in case of high drift; (iv) summation of consecutive frames to ensure a suitable tilt step for data processing. After pre-processing, the images were transformed into .pck format (Abrahams, 1993 ▸) for data processing using *XDS* (Kabsch, 2010 ▸).

### 2.6. Data processing   

Data were analysed with *XDS* (Kabsch, 2010 ▸), *POINTLESS*/*AIMLESS* (Winn *et al.*, 2011 ▸) and *XPREP* (Bruker, 2004 ▸). For analysis with *XDS*, single frames were summed to create a data set with a rotational increment of approximately 0.1° per frame. The rotation axis was estimated from the diffraction image assuming that reflections with wide rocking curves are close to the rotation axis. The camera length was estimated from the powder diffraction of an evaporated aluminium standard grid with known *d* spacings. The short wavelength of λ = 0.02508 Å leads to a very small 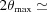 1.76° so that uncertainties in the camera length have a larger effect on the uncertainty of the unit-cell parameters (see Fig. S1 in the supporting information). In order to stabilize refinement of the experimental parameters, the Laue group was constrained to monoclinic, *i.e.* 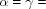 90°, and the camera length was not further refined.

The high-resolution cut-off was difficult to judge due to low completeness and low-symmetry space groups. We cut the resolution very roughly at 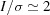 and 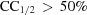 (Karplus & Diederichs, 2012 ▸).

Single-crystal data from carbamazepine and nicotinic acid were also processed with *ADT3D* (Kolb *et al.*, 2007 ▸, 2011 ▸)/*PETS* (Palatinus, 2011 ▸). Results are shown in Table S7.

### 2.7. Structure solution and refinement   

The structures were solved *ab initio* with *SHELXT* (Sheldrick, 2015 ▸); subsequent refinement of the given structure was done with *SHELXL* and *ShelXle* (Sheldrick, 2008 ▸; Hübschle *et al.*, 2011 ▸). The eight-parameter fitting for electron scattering factors from Peng (1999 ▸) was used for *SHELXL*.

The carbamazepine structure was refined anisotropically unrestrained except for the RIGU restraint (Thorn *et al.*, 2012 ▸). Nicotinic acid was refined isotropically without any restraints. Hydrogen atoms were added in riding positions. This results in 163 parameters for carbamazepine including the scaling factor between 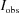 and 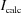 with 144 restraints, and 39 parameters with no restraints for nicotinic acid.

The structure of carbamazepine was also solved with *SIR2014* (Burla *et al.*, 2015 ▸) against the single-crystal data. Results are shown in Table S7.

## 3. Results   

**Figure 3:**
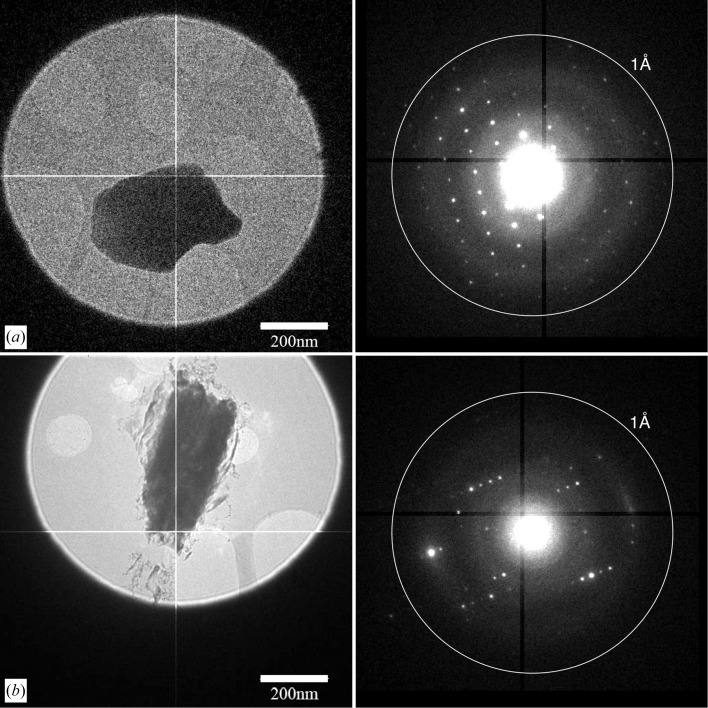
Rotation electron diffraction of pharmaceutical nanocrystals showing in the left panel an image of the crystal at 5.3k times magnification and in the right panel a diffraction pattern: (*a*) a 200 nm-thin carbamazepine crystal, (*b*) a 200 nm-thin nicotinic acid crystal.

Rotation ED data were collected at room temperature with a normal single tilt rotation holder of two different beam-sensitive pharmaceutical compounds (carbamazepine and nicotinic acid), using a CM30 microscope. Figs. 3 ▸(*a*) and 3 ▸(*b*) show crystals of both compounds. The CM30 had a modified flange which allowed fitting the housing of the Timepix detector in an on-axis position (Fig. 1 ▸). Stability of the goniometer and specimen holder limited the maximum range of continuous rotation at slightly more than 50° in some of the experiments. The Timepix detector was sufficiently sensitive to allow collection of high-quality, low-dose ED data of nanocrystals of these compounds for more than 5 min without any observable beam damage.

Both crystals diffracted beyond 1 Å resolution (Fig. 3 ▸). The data were of sufficiently high quality for both the structures of carbamazepine and nicotinic acid to be solved with direct methods. Given the uncertainty in the detector distance, the incomplete data and the different temperatures at which the data presented here and the literature values were collected, our results as shown in Tables 1 ▸, S1–S6 show remarkable agreement between our refined structures and published coordinates (El Hassan *et al.*, 2013 ▸; Kutoglu & Scheringer, 1983 ▸).

**Figure 4:**
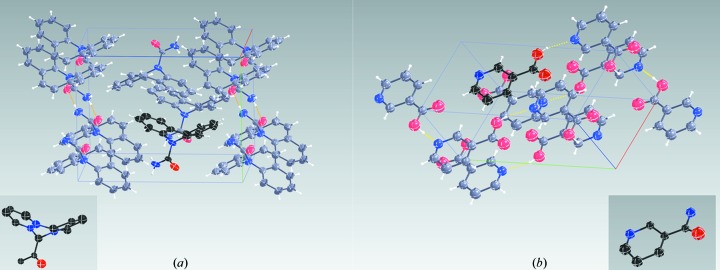
Unit-cell content for the final refined structures of carbamazepine (*a*) and nicotinic acid (*b*) for the single-crystal data set, Table 1 ▸. Insets show the *SHELXT* solutions.

Fig. 4 ▸ shows the unit-cell content of the refined structures of carbamazepine (Fig. 4 ▸ *a*) and nicotinic acid (Fig. 4 ▸ *b*) against the single-crystal data sets. The respective insets show the results from structure solution with *SHELXT* (see the supporting information for *R*1 values). Note that neither were additional atoms found, nor were any atoms missing after structure solution, and only a few atom types needed correction.

### 3.1. Carbamazepine   

The carbamazepine nanocrystals were thinner than 200 nm and their ED patterns showed Bragg spots to 0.8 Å resolution. In the low-dose conditions that were used, the crystals diffracted at room temperature for more than 5 min to this resolution without visible deterioration. They could be kept within the beam for more than 50° of continuous rotation without needing intermediate realignment. Data were collected at a frame rate of 9.1 fps whilst maintaining a constant rotation speed. The total data acquisition time for any data set did not exceed 5 min.

Data from a crystal of carbamazepine were collected with 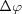 = 0.018° per frame and a total tilt of 51°. For data integration, six adjacent frames were summed, creating frames with angular width of 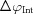 = 0.108° per frame. Indexing with *XDS* suggested a primitive monoclinic unit cell with cell parameters *a* = 7.53, *b* = 11.14, *c* = 14.06 Å and β = 92.80° (Table 1 ▸). These parameters are consistent with carbamazepine polymorph III (El Hassan *et al.*, 2013 ▸) measured by single-crystal X-ray diffraction, which were *a* = 7.49, *b* = 11.04, *c* = 13.77 Å and β = 94.4°, using a crystal with a volume that was ten orders of magnitude larger than the crystal we used. With this single data set we could already solve the structure with *SHELXT* (Sheldrick, 2015 ▸). Despite a light atom structure, *SHELXT* assigned all but three atom types correctly, did not miss any atoms and did not assign false peaks (see inset Fig. 4 ▸ *a*). Subsequent structure refinement with *SHELXL* (Sheldrick, 2008 ▸) was stable even without any geometric restraints, as shown in Tables S3 and S4 in the supporting information.

### 3.2. Nicotinic acid   

Continuous ED data were collected from a 200 nm-thin nicotinic acid crystal. The whole tilt range spanned 36°, with the tilt range from −26° to 10° with a frame width 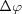 = 0.048°. For integration two frames were summed to 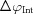 = 0.096° per frame. Indexing the diffraction patterns using *XDS* indicated a primitive monoclinic lattice and unit-cell parameters consistent with those from X-ray data collected at 100 K (Kutoglu & Scheringer, 1983 ▸) (see Table 1 ▸). Tables S5 and S6 in the supporting information summarize the good agreement between the structural model derived from these data and the published structure (Kutoglu & Scheringer, 1983 ▸).

## 4. Discussion   

We collected and processed data from two small-molecule compounds, carbamazepine and nicotinic acid, by electron diffraction from single nanometre-sized crystals at room temperature. We used a Timepix ASIC detector with a sensitivity about ten times higher than conventional CCD cameras. This detector, with a very fast readout, radiation hardness, sensitivity and which only integrates high-energy electron impacts, enabled data collection to high resolution of these radiation-sensitive organic samples at very low dose without observable sample degradation (∼4.0 e^−^ Å^−2^ for the carbamazepine data set and ∼1.1 e^−^ Å^−2^ for the nicotinic acid data set). Both data sets were of sufficient quality for structure solution by direct methods. To our knowledge, this is the first time that crystal structures of organic nanocrystals could be solved with direct methods using ED rotation data of single nanocrystals collected at room temperature using such a low total electron dose. The experimental limitation, due to the limited precision of the transmission electron microscope specimen stage and the holder stability, severely compromised data quality (see Table 1 ▸). Yet, in both cases unit-cell parameters and the refined structures are consistent with literature values based on X-ray structure analysis. We show that our technique of ultra-fast three-dimensional electron diffraction coupled with a sensitive Timepix detector allows fast and efficient three-dimensional crystal structure analysis of organic pharmaceutical compounds at room temperature. The technique will allow higher-throughput examination of nanometre-sized samples in a transmission electron microscope at room temperature and can be complementary to standard single-crystal and powder X-ray diffraction. Future improvement of transmission electron microscope goniometer stability for ED work and application of precession electron diffraction during data acquisition for precise atomic coordinates determination (Gemmi & Oleynikov, 2013 ▸) may extend the applications of electron crystallography to the wider TEM/X-ray scientific community. The techniques and methods shown here should pave the way to solving compounds with a larger unit cell at non-cryo conditions and the possibility of solving the structures of even more beam-sensitive protein nanocrystals (Nederlof *et al.*, 2013 ▸).

## 5. Conclusions   

Electron diffraction data were recorded with the new Timepix ASIC detector in conventional rotation mode. The data quality was good enough to solve two organic crystal structures with direct methods. We describe the advantages of our approach, in particular the detector type that is well suited for the recording of electron diffraction data. Based on the experience from this project, we are improving the instrumental design of both detector and diffraction instrument. We are also currently investigating how to produce better quality data. This will include processing programs dedicated to electron diffraction data and programs that take the effects of dynamic scattering into account.

## Supplementary Material

Crystal structure: contains datablock(s) carbamazepine_single, nicotinic_acid_single, carbamazepine_5x-merged, nicotinic_acid_2x-merged, carbamazepine_adt3d. DOI: 10.1107/S2053273315022500/td5026sup1.cif

Supporting information file. DOI: 10.1107/S2053273315022500/td5026sup2.pdf

Structure factors: contains datablock(s) carbamazepine_single. DOI: 10.1107/S2053273315022500/td5026sup3.hkl

Structure factors: contains datablock(s) nicotinic_acid_single. DOI: 10.1107/S2053273315022500/td5026sup4.hkl

Structure factors: contains datablock(s) carbamazepine_5x-merged. DOI: 10.1107/S2053273315022500/td5026sup5.hkl

Structure factors: contains datablock(s) nicotinic_acid_2x-merged. DOI: 10.1107/S2053273315022500/td5026sup6.hkl

Structure factors: contains datablock(s) carbamazepine_adt3d. DOI: 10.1107/S2053273315022500/td5026sup7.hkl

Movie from the carbamazepine data set. DOI: 10.1107/S2053273315022500/td5026sup8.avi

Movie from the nicotinic acid data set. DOI: 10.1107/S2053273315022500/td5026sup9.avi

CCDC references: 1438802, 1438803, 1438804, 1438805, 1438806

## Figures and Tables

**Table 1:** Data statistics from *XDS* and model *R* values after refinement with *SHELXL*

	Carbamazepine	Nicotinic acid
Molecular formula	C_15_H_12_N_2_O	C_6_H_5_NO_2_
Crystal size (µm)	1.2 × 0.8 × 0.2	0.7 × 1.6 × 0.2
Tilt range (°)	51.00	36.00
Δφ (° per frame)	0.018	0.048
Δφ_Int_ † (° per frame)	0.108	0.096
Dose (e^−^ Å^−2^)	4.0	1.1
Space group	*P*2_1_/*n*	*P*2_1_/*c*
Cell dimensions‡		
*a* (Å)	7.487 (1)	7.186 (2)
*b* (Å)	11.041 (2)	11.688 (3)
*c* (Å)	3.775 (3)	7.231 (2)
β (°)	92.94 (4)	113.55 (6)
		
Data processing		
Cell dimensions		
*a* (Å)	7.53 (1)	7.30 (1)
*b* (Å)	11.139 (6)	11.693 (2)
*c* (Å)	14.06 (2)	7.33 (3)
β (°)	92.80 (8)	113.7 (1)
Resolution§ (Å)	8.73–0.8 (0.85–0.80)	5.82–0.75 (0.86–0.75)
*R*_merge_ ¶ (%)	8.4 (35.8)	7.1 (34.9)
*I*/σ	5.64 (1.80)	4.96 (1.75)
CC_1/2_ ††	99.0 (53.0)	99.4 (83.1)
Completeness (%)	45.0 (46.2)	35.6 (36.0)
Reflections	2202 (371)	953 (152)
Unique reflections	1071 (181)	503 (82)
		
Refinement statistics		
*R*_complete_ ‡‡	32.2	37.9
*R*1 (%)	28.0	35.6
*wR*2 (%)	55.6	63.9

† Frames were summed before integration.

‡ El Hassan *et al.* (2013 ▸) for carbamazepine and Kutoglu & Scheringer (1983 ▸) for nicotinic acid.

§ Values in parentheses here and below denote the highest-resolution shell.

¶ *R*_merge_ ≡ *R*_sym_ ≡ *R*_int_ = 
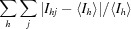
.

†† Karplus & Diederichs (2012 ▸).

‡‡ Luebben & Gruene (2015 ▸).

## Footnotes

1 In this paper, we abbreviate ‘nanometre-sized crystals’ to nanocrystals.

2 We speak of ‘camera’ for the whole assembly of detector, readout and pod parts. ‘Detector’ is used for the Timepix chip including the sensitive layer.

3 At the final steps the beam was acquired on the Timepix detector because the sensitivity of the fluorescent screen was no longer strong enough to visualize the electrons.
