# *Ab initio* structure determination of nanocrystals of organic pharmaceutical compounds by electron diffraction at room temperature using a Timepix quantum area direct electron detector. Corrigendum

**DOI:** 10.1107/S2053273318014079

**Published:** 2018-10-30

**Authors:** E. van Genderen, M. T. B. Clabbers, P. P. Das, A. Stewart, I. Nederlof, K. C. Barentsen, Q. Portillo, N. S. Pannu, S. Nicolopoulos, T. Gruene, J. P. Abrahams

**Affiliations:** a Biophysical Structural Chemistry, Leiden University, Einsteinweg 55, 2333 CC Leiden, The Netherlands; b Center for Cellular Imaging and NanoAnalytics (C-CINA), Biozentrum, University of Basel, CH-4058 Basel, Switzerland; c Nanomegas SPRL, Boulevard Edmond Machtens 79, B 1080, Brussels, Belgium; d Department of Physics and Energy, Materials and Surface Science Institute (MSSI), University of Limerick, Limerick, Ireland; e Amsterdam Scientific Instruments, Postbus 41882, 1009 DB Amsterdam, The Netherlands; f Centres Científics i Tecnològics de la Universitat de Barcelona, University of Barcelona, Carrer de Lluís Solé i Sabaris, 1-3, Barcelona, Spain; g Biology and Chemistry, Laboratory of Biomolecular Research, Paul Scherrer Institute (PSI), 5232 Villigen, Switzerland

**Keywords:** electron nanocrystallography, Timepix quantum area detector, carbamazepine, nicotinic acid, electron diffraction structure determination

## Abstract

Corrections are made to Table 1 in the article by van Genderen *et al.* [*Acta Cryst*. (2016), A**72**, 236–242].

## Abstract 2

A correction is made to the article by van Genderen *et al.* [*Acta Cryst*. (2016), A**72**, 236–242].

In the article by van Genderen *et al.* (2016 ▸), the scattering factors used in the refinements and in the CIF file were for X-ray scattering rather than for electron scattering. The correct scattering factors have now been used and the statistics that were affected by this error (model statistics *R*_complete_, *R*1 and *wR*2) have been recalculated.

This affects six entries in Table 1 ▸ of the original publication. The correct values are given here. The corrected CIF and supporting information are also made available.

## Supplementary Material

Crystal structure: contains datablock(s) global, carbamazepine_single, carbamazepine_5x-merged, nicotinic_acid_single, nicotinic_acid_2x-merged. DOI: 10.1107/S2053273318014079/td9026sup1.cif

Supporting information file. DOI: 10.1107/S2053273318014079/td9026sup2.pdf

CCDC references: 1438802, 1438803, 1438804, 1438805, 1438806

## Figures and Tables

**Table 1:** Corrected statistics

	Carbamazepine	Nicotinic acid
Refinement statistics		
*R*_complete_ †	31.8	37.7
*R*1 (%)	27.9	34.1
*wR*2 (%)	55.2	60.1

† Luebben & Gruene (2015 ▸).
