# Supplementary material for: Ab initio structure determination of nanocrystals of organic pharmaceutical compounds by electron diffraction at room temperature using a Timepix quantum area direct electron detector
Source: Acta Crystallogr A Found Adv. 2016 Feb 5;72(Pt 2):236–42. doi: 10.1107/S2053273315022500 (PMC4770873; doi:10.1107/S2053273315022500)
Supplement: Supplementary file 2 [file a-72-00236-sup2.pdf]

Ab initio structure determination of nanocrystals of organic pharmaceutical compounds by electron diffraction at room temperature using a Timepix quantum area direct electron detector — Supplemental Material —

E. van Genderen, M.T.B. Clabbers, P.P. Das, A. Stewart,
I. Nederlof, K.C. Barentsen, Q. Portillo, N.S. Pannu, S. Nicolopoulos, T. Gruene*, J.P. Abrahams*

January 14, 2016

Acta Crystallographica Section A72 (2016)
doi:10.1107/S2053273315022500

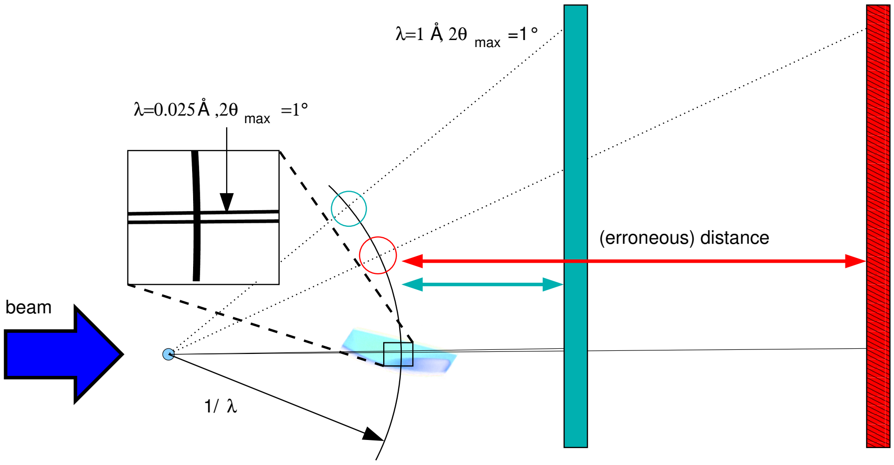

Figure S1: A short wavelength leads to greater errors in the unit cell parameters. At short wavelengths typical to electron diffraction, $\lambda = 0.0251$Å, the maximum diffraction angle 2θ is very small. See text for details.

S1 Camera Length and Cell Parameters

The wavelength used for electron diffraction is typically about $\lambda \approx 0.025$Å, whereas in X–ray diffraction, it is about $\lambda \approx 0.5$–1.5Å. With a maximum resolution of 1Å, common for crystals from organic, the short wavelength leads to a small maximum diffraction angle, $2\theta_{\text{max}} \approx 1$-2°, as opposed to $2\theta_{\text{max}} \approx 40$–170° in X–ray diffraction. In order to calculate the reciprocal lattice, the recorded diffractions are projected onto the Ewald sphere. At short wavelength, even a threefold greater camera lengths leads to only small distortions of the lattice. Therefore, uncertainties in the detector distance have much greater effect on the accuracy of the unit cell parameters. This is illustrated in Fig. S1.

S2 Structure Solution

Data processed with XDS were solved with SHELXT. As we could not use the default options, we list the options for each data set here together with a summary of the results:

Carbamazepine, Single Crystal (Table 1)

```
$ shelxt cbz_a005 -a -z1.5 -m1000 -k10 -q0 -s"P2(1)_n"
   R1   Rweak Alpha    Space group  Flack_x  Formula
 0.352 0.296 0.281    P2(1)/n               C14 N3 O
```

Carbamazepine, Merged Data from five crystals (Table S1)

```
$ shelxt cbz_mrg -a -z1.5 -m1000 -k10 -q0 -s"P2(1)_n"
   R1  Rweak Alpha   Space group  Flack_x  Formula
 0.452 0.248 0.422   P2(1)/n               C11 N6 O2
```

Nicotinic Acid (Table 1)

```
$ shelxt na_b004 -a -m1000
   R1  Rweak Alpha   Space group  Flack_x  Formula
 0.378 0.078 0.150   P2(1)/c               C6 N2 O
 0.382 0.047 0.096   Pc            -4.21   C7 N O4
 0.346 0.057 0.134   P2(1)         -2.29   C11 N O4
```

Nicotinic Acid, Merged data from two crystals (Table S2)

```
$ shelxt na_rg -a -m1000
   R1  Rweak Alpha   Space group  Flack_x  Formula
   0.460 0.390 0.184 P2(1)/c               C5 N3 O
   0.410 0.167 0.068 P2(1)         no Fp   C10 N2 O7
   0.441 0.198 0.220 Pc            no Fp   C9 N4 O3
```

S3 Merging Data

The main article shows data statistics for single data sets from carbamazepine and from nicotinic acid. We collected additional data sets from five different crystals for carbamazepine and two different crystals for nicotinic acid in order to see how merging affects the data. The data are shown in Table S1 and Table S2 respectively. The merging statistics in both cases are worse than for the single data sets, and structure solution with *SHELXT* required more options (see Sec. S2. It is not clear whether this is due to the effect of dynamic scattering. The amide group of carbamazepine can be oriented in two ways. During structure refinement against a data set of a single crystal we could observe a difference in $R1$ between both possibilities, so that one crystal appears to crystallize with a preferred orientation. Several crystals may be a mixture of either orientation which could be a reason for the poor statistics. However, this is only a hypothesis. As this question is not the central question of this work, we did not investigate the effect at this stage.

Data were merged using *POINTLESS/AIMLESS* [1] as merging with XSCALE produced worse results. We are currently investigating the reasons. Note that *AIMLESS* has more strict rejection criteria than XSCALE, hence the sum of reflections in Table S4 from each crystal is greater than the total number of reflections for the merged data set. *POINTLESS* calculates a weighted averaged cell from the unit cell parameters.

Table S1: Data processing statistics of XDS from five different carbamazepine crystals and *R*–values from refinement with SHELXL. Res: resolution range, high resolution shell in brackets where applicable. Compl: % Completeness. Refl: Total number of reflections.

	Crystal 1	Crystal 2	Crystal 3	Crystal 4	Crystal 5	5x merged
ϕ_{total} [°]	50	38.8	37.1	40	41	
$\Delta\phi$	0.039	0.016	0.012	0.022	0.031	
$\Delta\phi_{\text{Int}}$[1]	0.117	0.098	0.098	0.088	0.093	
Cell						
a	7.58	7.58	7.58	7.55	7.59	7.58
b	11.17	11.20	11.19	11.17	11.21	11.20
c	13.99	13.93	13.93	13.96	14.03	13.93
β	93.1	92.7	93.0	92.7	93.4	92.7
Res. [Å]	8.73.0–0.81	11.20–0.81	8.72–0.91	7.54–0.83	11.21–1.16	11.19 – 0.81
	(0.87–0.81)	(0.86–0.81)	(0.96–0.91)	(0.88–0.83)	(1.23–1.16)	(0.87–0.81)
R_{merge} [%]	6.7 (37.2)	8.3 (36.4)	8.1 (38.5)	7.0 (22.1)	21.9 (42.0)	16.2 (50.8)
I/σ	6.18 (1.74)	7.05 (1.79)	5.91 (1.80)	7.96 (1.84)	3.09(1.99)	7.0 (1.8)
$CC_{1/2}$	99.4 (62.2)	99.5 (76.5)	99.3 (69.3)	99.4 (82.5)	95.7 (63.1)	99.0 (84.3)
Compl	45.1 (48.6)	26.2 (27.0)	28.9 (26.9)	17.9(3.7)	27.4 (29.4)	77.5 (62.3)
Refl.	1950	1685	1364	1402	1617	5415 (456)
Unique	1066	607	668	654	615	1861 (237)
R_{complete}						45.6
R1 [%]						43.2
wR2 [%]						71.1

Table S2: Data processing statistics of XDS from two different nicotinic acid crystals and *R*–values from refinement with SHELXL. Data for "Crystal 1" identical with nicotinic acid entry in Table 1. n/a: not applicable; n/c: not computed

	Crystal 1	Crystal 2	2x merged
Tilt range [°]	36.0	36.2	n/a
$\Delta\phi$ [°/frame]	0.048	0.021	n/a
$\Delta\phi_{\mathrm{Int}}{}^2$ [°/frame]	0.096	0.107	n/a
Cell			
a	7.30(1)	7.42(1)	7.406
b	11.693(2)	11.690(7)	11.692
c	7.33(3)	7.509(7)	7.377
β	113.7(1)	115.21(5)	114.45
Resolution [Å]	5.82–0.75	11.70–1.16	11.69–0.75
	(0.86–0.75)	(1.23–1.16)	(0.84–0.75)
R_{merge} (%)	7.1 (34.9)	11.2 (34.8)	21.0 (32.9)
I/σ	4.96 (1.75)	3.90 (1.85)	4.7 (1.9)
$CC_{1/2}$	99.4 (83.1)	98.9 (92.7)	97.6 (85.9)
Completeness (%)	35.6 (36.0)	34.8 (29.1)	38.6 (37.9)
Reflections	953 (152)	304 (31)	1266 (274)
Unique	503 (82)	139 (16)	542 (147)
R_{complete}	37.9	n/c	44.2
R1 [%]	35.6	n/c	40.2
wR2 [%]	63.9	n/c	69.7

S4 Model Quality

In order to assess the data quality beyond the statistical descriptors in Tables 1 and 2, a comparison of the bond lengths and angles for carbamazepine with the values published by [2] is shown in Tables S3 and S4. All structures were refined without restraints. Standard uncertainties as provided by SHELXL.

Table S3: Bond lengths for carbamazepine in comparison with the high resolution structure by [2]. *single*: single crystal data set. Table 1; *5x merged*: merged data from five crystals, Table S1. Lengths listed in [Å].

	El Hassan	*single*	*5x merged*
N1-C15	1.3815(5)	1.42(3)	1.37(2)
N2-C15	1.3566(4)	1.37(3)	1.39(2)
C15-O1	1.2384(4)	1.19(3)	1.24(2)
N1-C1	1.4302(4)	1.40(4)	1.45(2)
N1-C14	1.4316(4)	1.41(3)	1.43(2)
C9-C14	1.4041(4)	1.41(2)	1.42(3)
C1-C6	1.4054(4)	1.49(3)	1.38(3)
C8-C9	1.4624(5)	1.45(4)	1.45(2)
C6-C7	1.4619(5)	1.55(3)	1.45(3)
C7-C8	1.3520(6)	1.24(4)	1.34(3)
C5-C6	1.4080(4)	1.37(4)	1.35(3)
C4-C5	1.3883(5)	1.38(4)	1.43(3)
C3-C4	1.3966(6)	1.33(3)	1.36(3)
C2-C3	1.3930(6)	1.46(4)	1.38(3)
C1-C2	1.3959(5)	1.36(2)	1.44(3)
C9-C10	1.4063(5)	1.46(3)	1.40(3)
C10-C11	1.3882(6)	1.36(5)	1.35(3)
C11-C12	1.3959(6)	1.33(3)	1.41(3)
C12-C13	1.3936(6)	1.42(3)	1.38(3)
C13-C14	1.3988(5)	1.49(4)	1.43(2)

S5 Data Processing and Structure Solution with ADT3D / PETS and SIR2014

For analysis with *ADT3D* [4]/*PETS* [5], adjacent frames were summed to ensure a rotation range of $\approx 1.0°$ per frame. Background correction and centering of the diffraction pattern with respect to the direct beam was performed. First, the rotation axis was determined using *ADT3D*/*PETS*, then the 3D reciprocal lattice vector space was reconstructed using *ADT3D*/*PETS*, allowing unit cell determination. Using the unit cell parameters, reflections were automatically indexed and

Table S4: Selected angles for carbamazepine in comparison with the high resolution structure by [2]. Angles in °. Column headers as in Table S3.

	El Hassan	*single*	*5x merged*
C1-N1-C14	117.13(3)	119(1)	116(1)
O1-C15-N2	122.26(3)	122(1)	120(1)
C7-C8-C9	126.25(3)	128(2)	129(2)
C6-C7-C8	127.75(3)	126(2)	126(2)
C8-C9-C14	123.04(3)	124(2)	124(2)
C1-C6-C7	123.44(3)	119(2)	125(2)

Table S5: Bond lengths of nicotinic acid in comparison with the high resolution structure by [3]. *single*: single crystal data set. Table 1; *2x merged*: merged data from two crystals, Table S2. Lengths listed in [Å].

	Kutoglu	*single*	*2x merged*
N-C	1.348(4)	1.36(2)	1.41(3)
C1-C2	1.397(4)	1.41(1)	1.46(3)
C2-C3	1.406(4)	1.44(4)	1.50(5)
C3-C4	1.383(5)	1.42(3)	1.33(3)
C4-C5	1.393(5)	1.41(3)	1.36(4)
C5-N	1.342(4)	1.28(4)	1.26(5)
C2-C6	1.490(4)	1.46(2)	1.53(3)
C6-O1	1.308(4)	1.34(3)	1.32(5)
C6-O2	1.211(4)	1.16(2)	1.14(3)

Table S6: Selected angles for nicotinic acid in comparison with the high resolution structure by [3]. Angles in °. Column headers as in Table S5.

	Kutoglu	*single*	*2x merged*
C1-N1-C14	117.13(3)	119(1)	116(1)
O1-C15-N2	122.26(3)	122(1)	120(1)
C7-C8-C9	126.25(3)	128(2)	129(2)
C6-C7-C8	127.75(3)	126(2)	126(2)
C8-C9-C14	123.04(3)	124(2)	124(2)
C1-C6-C7	123.44(3)	119(2)	125(2)

Table S7: Statistics of data processing by *ADT3D/PETS* and model *R*–values for carbamazepine after refinement with *SIR2014*. Model *R*–values are not available for nicotinic acid, marked n/a. Differences in the presented statistics between this table and Table 1 are due to differences in the software used.

	Carbamazepine	Nicotinic acid
Tilt range [°]	51.00	36.00
$\Delta\phi$ [°/frame]	0.018	0.048
$\Delta\phi_{\mathrm{Int}}{}^3$ [°/frame]	0.990	1.008
Space group	$P2_1/n$	$P2_1/c$
Cell dimensions		
a, b, c [Å]	7.68, 11.44, 13.92	7.19, 11.74, 7.28
α, β, γ [°]	89.39, 91.22, 89.43	89.62, 112.45, 90.35
Resolution [Å]	8.84–0.69	5.84–0.71
R_{merge} [%]	13.58	31.44
$F/\sigma(F)$	15.03	1.58
Completeness (%)	50.16	38.0
Reflections	3868	1047
Unique reflections	1940	618
R1 (%)	38.9	n/a
wR2 (%)	36.9	n/a

intensities extracted. For intensity extraction, no further corrections (like Lorentz polarization) were performed. The extracted intensities allowed ab initio structure solution using direct methods implemented in *SIR2014* for the case of carbamazepine and simulated annealing technique for nicotinic acid compound. For structure solution, the kinematical approximation was used, where $I \propto F^2(hkl)$, which proved to be sufficient.

ADT3D integrates reflections from the entire frames. The resolution cut–off was subsequently chosen by *SIR2014* based on the useful data.

S5.1 Carbamazepine

Using *ADT3D* / *PETS* we analyzed the same rotation diffraction data of carbamazepine processed with *XDS* having a tilt range of $-25°$ – $26°$, see Table 1. For data processing, blocks of 55 adjacent frames were summed to create a data set with an angular increment of $\approx 1.0°$ per frame, which was required for *ADT3D*. The obtained unit cell parameters ($a = 7.68$ Å, $b = 11.44$ Å, $c = 13.92$Å, $\alpha = 89.39$, $\beta = 91.22°$, and $\gamma = 89.43$) are consistent with the literature values [2]. A total number of 3868 reflections were extracted from the collected data, after applying the (2/m) monoclinic Laue symmetry 1940 reflections were left over.

The space group $P2_1/n$ was automatically recognized by *SIR2014* (figure of merit 0.88). Structure solution for carbamazepine was possible in one step using direct methods (*SIR2014*) with an initial residual value of 34.91% with 4 ghost atoms, 2 C atoms were assigned as N, O/N

atoms were not assigned and after the refinement the $R1$ value reaches to 38.9%. During refinement rigid body restraint was used for one of the benzene rings and all the atoms were refined isotopically.

A comparison of bond lenghts and selected 1,3–angles with literature values is presented in Tables S8 and S9 respectively.

Table S8: Bond lengths for carbamazepine refined with *SIR2014* against data processed with *ADT3D / PETS* in comparison with the high resolution structure by [2]. See also Table S7. Lengths listed in [Å].

	El Hassan	*adt3d*
N1-C15	1.3815(5)	1.595
N2-C15	1.3566(4)	1.380
C15-O1	1.2384(4)	1.182
N1-C1	1.4302(4)	1.379
N1-C14	1.4316(4)	1.484
C9-C14	1.4041(4)	1.458
C1-C6	1.4054(4)	1.307
C8-C9	1.4624(5)	1.433
C6-C7	1.4619(5)	1.468
C7-C8	1.3520(6)	1.268
C5-C6	1.4080(4)	1.432
C4-C5	1.3883(5)	1.672
C3-C4	1.3966(6)	1.432
C2-C3	1.3930(6)	1.606
C1-C2	1.3959(5)	1.531
C9-C10	1.4063(5)	1.482
C10-C11	1.3882(6)	1.592
C11-C12	1.3959(6)	1.373
C12-C13	1.3936(6)	1.407
C13-C14	1.3988(5)	1.511

S5.2 Data Processing for Nicotinic Acid

Continuous rotation data was collected using a 200 nm thin nicotinic acid nanocrystal. The whole tilt range spanned 36° in total, with the tilt range from $-26°$–10° with an angular increment of 0.05° (see Table 1). Data processing with *ADT3D* required summing 21 adjacent frames for 1° tilt. The unit cell parameters (a = 7.19Å, b = 11.74Å, c = 7.28Å, α = 89.62, β = 112.45°, and γ = 90.35) are consistent with the literature values [3]. After intensity extraction based on the obtained unit cell, the total number of reflections was 1032 with a resolution of up to 0.78°Å. The space group $P2_1/c$ was suggested by *SIR2014* with a figure of merit 0.137. However, *ab initio* structure solution using *SIR2014* was not successful as the data completeness

Table S9: Selected angles for carbamazepine refined with *SIR2014* against data processed with *ADT3D* / *PETS* in comparison with the high resolution structure by [2]. Angles in °. Column headers as in Table S8.

	El Hassan	*single*
C1-N1-C14	117.13(3)	120.6
O1-C15-N2	122.26(3)	129.5
C7-C8-C9	126.25(3)	131.1
C6-C7-C8	127.75(3)	129.5
C8-C9-C14	123.04(3)	123.1
C1-C6-C7	123.44(3)	119.4

was too low (38%) due to limited tilt range. As an alternative we have used simulated annealing (SA) implemented in *SIR2014* to solve the crystal structure and results obtained so far will be published elsewhere.

References

[1] Winn, M. D. et al. *Acta Cryst.* **2011**, *D67*, 235–242.

[2] El Hassan, N.; Ikni, A.; Gillet, J.-M.; Spasojevic-de Biré, A.; Ghermani, N. E. *Crystal Growth & Design* **2013**, *13*, 2887–2896.

[3] Kutoglu, A.; Scheringer, C. *Acta Cryst.* **1983**, *C39*, 232–234.

[4] Kolb, U.; Gorelik, T. E.; Kübel, C.; Otten, M. T.; Hubert, D. *Ultramicroscopy* **2007**, *107*, 507–513.

[5] Palatinus, L. PETS — program for analysis of electron diffraction data. Prague: Institute of Physics of the AS CR, 2011.
